# The hepcidin‐ferroportin axis influences mitochondrial function, proliferation, and migration in pulmonary artery endothelial and smooth muscle cells

**DOI:** 10.1002/pul2.70006

**Published:** 2024-12-18

**Authors:** Theo Issitt, Quezia K. Toe, Sofia L. Pedersen, Thomas Shackshaft, Maziah Mohd Ghazaly, Laura West, Nadine D. Arnold, Abdul Mahomed, George W. Kagugube, Latha Ramakrishnan, Allan Lawrie, Gregory J. Quinlan, S. John Wort

**Affiliations:** ^1^ NHLI, Faculty of Medicine Imperial College London London UK; ^2^ Institute of Tropical Biodiversity and Sustainable Development Univerity of Malaysia Terengganu Malaysia; ^3^ Department of Infection, Immunity & Cardiovascular Disease University of Sheffield Sheffield UK; ^4^ Royal Brompton Hospital Adult Centre for Pulmonary Hypertension London UK

**Keywords:** hepcidin ferroportin axis, iron, mitochondria, pulmonary artery hypertension

## Abstract

Elevated circulating hepcidin levels have been reported in patients with pulmonary artery hypertension (PAH). Hepcidin has been shown to promote proliferation of human pulmonary artery smooth muscle cells (PASMCs) in vitro, suggesting a potential role in PAH pathogenesis. However, the role of human pulmonary artery endothelial cells (PAECs) as either a source of hepcidin, or the effect of hepcidin on PAEC function is not as well described. The objective of this study was to define the role of the hepcidin‐ferroportin axis on the phenotype of PAEC and to study potential PAEC‐PASMC interactions relevant to the pathogenesis of pulmonary vascular remodeling and PAH. PAECs treated with hepcidin, or interleukin‐6 were investigated for both ferroportin and hepcidin release and regulation using immunofluorescence, mRNA levels and cellular release assays. Effects of hepcidin on PASMC and PAEC mitochondrial function was investigated using immunofluorescence and seahorse assay. Migration and proliferation of PASMCs treated with conditioned media from hPAEC treated with hepcidin was investigated using the xCELLigence system and other tools. We demonstrate in this study that PAECs express ferroportin; hepcidin treatment of PAECs resulted in mitochondrial iron accumulation and intracellular hepcidin biosynthesis and release. Conditioned media from hepcidin treated PAECs caused PASMCs to down‐regulate ferroportin expression whilst promoting migration and proliferation. Inhibition of hepcidin in PAEC conditioned media limited these responses. PASMC cellular and mitochondrial iron retention are associated with migratory and proliferative responses. This study confirms that the hepcidin ferroportin axis is present and operational in PAECs. Modulation of this axis shows distinct differences in responses seen between PAECS and PASMCs. Stimulation of this axis in PAECs with hepcidin may well institute proliferative and migratory responses in PASMCs of relevance to pathogenesis of PAH offering potential novel therapeutic targets.

**Abbreviations**
IL‐6: Interleukin‐6
PASMCs: Human Pulmonary artery smooth muscle cells
PAECs: Human pulmonary artery endothelial cells
PAH: pulmonary artery hypertension

## INTRODUCTION

Pulmonary artery hypertension (PAH) is a rare, life‐limiting disease with limited treatments options. Remodeling of resistance, precapillary, pulmonary blood vessels including sustained vasoconstriction and the proliferation of pulmonary artery smooth muscle cells (PASMCs) and endothelial cells (PAECs), results in vessel wall muscularization, lumen restriction and eventually obliterative lesions. The subsequent elevation in pulmonaryvascular resistance leads to right sided heart failure and early death.^1^

Several genes and genetic abnormalities have been identified in the heritable form of PAH with mutations in the bone morphogenetic protein receptor (BMPR) II the most common.^2^, ^3^ Moreover, deficiency in BMPRII protein levels (and associated signaling molecules) has been shown in nongenetic and other forms of PAH cases,^4^ suggesting commonality, and potentially offering some mechanistic insight. A role for other members of the transforming growth factor beta (TGF‐β) super family of receptors has also been implicated in PAH.^5^

Signaling molecules in the TGF‐β superfamily including BMP/SMAD pathways are crucial for cell turnover and maintaining the balance between proliferation and apoptosis. In addition BMP/SMAD pathways are involved with numerous regulatory functions including that of iron homeostasis; specifically, through the control of the expression and release of the small peptide hormone, hepcidin,^6^ the master regulator of body iron turnover and control. In this regard, disrupted iron homeostatic control has been implicated in the pathogenesis of idiopathic PAH^7^ and related conditions, such as Eisenmenger syndrome.^8^ Hepcidin functions via interaction and downregulation of ferroportin activity, ferroportin being the only known cellular iron exporter in mammalian systems.^9^ Ferroportin expression is largely restricted to expression in hepatocytes, erythroblasts, enterocytes and sub‐types of macrophages, all cells with key function for iron regulation and reprocessing. However, ferroportin is also expressed more widely^9^, ^10^ including in human PASMCs where our group have shown that hepcidin treatment stimulates proliferation and cellular iron accumulation. Moreover, stabilization of ferroportin membrane localization and activity was able to reverse this proliferative response and iron accumulation despite the presence of hepcidin.^11^ Corroboration for the operation of the hepcidin‐ferroportin axis in PASMCs of relevance to PAH has recently been provided in vivo and in human cells,^12^ suggesting a localized iron regulatory axis may be operational in this vascular bed which may have implications for PASMC remodeling should homeostatic control be lost. Indeed, iron is a mitogen and cofactor for ribonucleotide reductase^13^ a key enzyme for DNA biosynthesis, and additionally for several cell cycle cyclins.^14^

Therefore, implications for altered iron regulation in any such cells will likely profoundly impact mitochondrial function given the key requirement of iron for respiration and biosynthesis within these organelles. Moreover, cellular iron‐loading is known to cause mitochondrial dysfunction, enhancing intracellular reactive oxygen species (ROS) production, affecting intracellular cell signaling responses and ultimately cell fate.^15^ Furthermore, throughout the literature, a role for mitochondrial dysfunction in PAH is emerging.^16^

This study investigates the hepcidin‐ferroportin axis in PAECs and PASMCs. We show that hepcidin exposure to PAECs and PASMCs causes strikingly different mitochondrial responses between the cell types with PASMCs showing pronounced mitochondrial dysfunction. In addition, hepcidin exposure to PASMCs promotes enhanced autocrine hepcidin production and release from these cells, and further that these processes stimulate both migration and proliferation in PASMCs. All these findings may be of relevance to further understanding the pathogenesis of vascular remodeling in PAH.

## METHODS

### Tissue and cell culture

Human PASMCs purchased from Promocell and ATCC were from four donors and maintained in Dulbecco's modified eagle's medium (DMEM) supplemented with 15% fetal calf serum (FCS), 2 mM glutamine, 100 U/mL penicillin, and 100 μg/mL streptomycin (Ramakrishnan et al. 2018). PASMC isolation and maintenance has been previously described by our group (Ramakrishnan et al. 2018) but briefly; PASMCs were isolated from samples of pulmonary artery from patients undergoing lung lobectomy at the Royal Brompton Hospital. To select only PASMCs for study from cells isolated from pulmonary arteries. PASMCs were selected in high serum DMEM over a very long period thereby excluding other cell types and characterized by morphological examination. Cells were characterized as described using smooth muscle cell markers smooth muscle actin, SM‐22 and SM‐MHC (Ramakrishnan et al. 2018). Cells were collected from seven donors over a blinded study.

All human PAECs from four donors (two male of ages 48 and 58 and two female of ages 34 and 55) were supplied fully characterized by Promocell and grown in endothelial cell basal medium 2 (ECM), with supplements and 2% FCS (Promocell). All cells were grown at 37°C in a humidified atmospheric incubator with 5% CO_2_. All experiments used at least three separate donors, and some donor specific responses were observed.

### Treatments and conditioned media preparation

Both PAECs and PASMCs were grown to a 90% confluent monolayer before trypsinization with the detach kit (Promocell) as per manufacturer's instructions. Before treatments, cells were starved overnight in serum restricted media (0.2% FCS containing ECM with supplements as supplied, excluding heparin and hydrocortisone or 0.2% FCS containing DMEM with 2 mM glutamine, 100 U/mL penicillin, and 100 μg/mL streptomycin). Treatment of cells with respective molecules was performed in serum restricted media. Hepcidin‐25 was purchased from Peptides International and interleukin‐6 (IL‐6) from R&D Systems.

For production of conditioned media, PAECs were seeded in 12‐well plates at a density of 7.5 × 10^4^ cells/well. Cultured cells were only used for experiments between passage 4 and 7. After starvation, cells were treated were serum restricted media (control), hepcidin at 1 µg/mL or IL‐6 at 10 ng/mL (unless otherwise specified). Following 24 h of exposure, media was collected and used immediately for direct treatment of PASMCs or flash frozen in dry ice and kept at −80°C.

### Transfection

PAECs were transfected with lipofectamine RNAiMAX (Thermofisher) transfecting agent with SMARTpool siRNA targeting hepcidin anitmicrobial peptide (HAMP) (Dharmacon cat#: L‐014014‐00‐0005) or control ON‐TARGETplus nontargeting control pool (Dharmacon Cat#: D‐001810‐10‐05).

### Gene expression

mRNA was collected from cultured cells using the RNAeasy system (Qiagen) and converted to cDNA using the first strand cDNA synthesis kit (Thermofisher) or iScript cDNA kit (Bio‐Rad) for total RNA synthesis. Amplification was performed with SYBR green reagents (Bio‐Rad).

### xCELLigence assay

For proliferation experiments with direct treatments, PASMCs were plated and starved as described in the respective treatment conditions. After 24 h, cells were treated with 0.2% FCS DMEM and no further additives (control) for 90 min at 37°C. After blocking, the PASMCs were treated with 15% FCS DMEM and no further additives (control), or hepcidin at 1 µg/mL or 100 ng/mL, or IL‐6 at 10 or 1 ng/mL. Repeat treatments were performed 48 h later.

Similar to the proliferation assay, the xCELLigence RTCA system can be used to measure cell migration in real time. The migration plates consist of wells separated into two connected chambers: cells are placed in the upper one, while conditioned media or other treatments are placed in the lower one. Between the two chambers, a microporous membrane allows cell migration. Migration is measured by the RTCA system as changes in electrical impedance; these occur as cells migrate and attach to the underside of the microporous membrane, which is covered in a gold microelectrode array.

160 µL conditioned media collected from PAECs was added to the lower chambers, as well as ECM and 15% FCS DMEM controls. The top chamber was then affixed to the lower chamber and 50 µL of 0.2% FCS DMEM added to each upper chamber. The apparatus was then incubated at 37°C for 1 h, after which a background reading of electrical impedance was taken. 100 µL PASMC solution was added to each upper chamber at a density of 15,000 cells/well. The plates were incubated at room temperature for 30 min to allow the cells to sediment before commencing RTCA Cell Index measurements every 15 min for 16 h.

### BRDU assay

Cells were plated at 70% confluence on 96 well plate (2500 cells/well). After initial adherence, the cells were serum starved (0.2% Serum) for 24 h before treatment. Following treatment for 24 h, BrdU (5‐bromo‐2'‐deoxyuridine) was introduced (at the manufacturer recommended concentration) for an additional 24 h incubation before harvesting the plates. Proliferation was quantified using anti‐BrdU‐POD antibody, according to the manufacturer's instructions. All treatments were undertaken in triplicate.

### MTS assays

Cell proliferation was analyzed by the CellTiter 96® Aqueous One Solution Cell proliferation Assay (Promega). Following cell treatments, the supernatant of each each well (96‐well plate) was removed and replaced with 100 μL of fresh complete medium and 20 μL of MTS assay was added. Three blank wells were also added, containing media and MTS assay only as a negative control.

### Immunoblotting

Cells were lysed in RIPA buffer (Sigma‐Aldrich) with protease and phosphatase inhibitor cocktails (Sigma‐Aldrich) for 10 min on ice. 40 µg of protein was resuspended in laemelli buffer denatured at 95°C for 5 min, separated by SDS‐PAGE and transferred to a nitrocellulose membrane. Blots were incubated over night at 4°C with primary antibodies for ferroportin (rabbit anti‐FPN [Sigma‐Aldrich]) and α‐tubulin (mouse anti‐α‐tubulin, Proteintech). HRP‐conjugated antibodies were used to visualize blots.

### Immunoflourescence

For experiments with fixed samples, PAECs and PASMCs were fixed in 4% paraformaldehyde in phosphate‐buffered saline (PBS) for 7 min, permeabilized in 0.25% Triton X‐100 in PBS for 5 min, blocked for 1 h in 0.1% BSA in PBS and incubated overnight with the following primary antibodies: Rabbit anti‐TOM20, (Proteintech), DAPI and rabbit anti‐FPN. The following conjugated secondary antibodies were used: Alexa‐488 conjugated goat anti‐rabbit/anti‐mouse, Alexa‐594 donkey anti‐rabbit/anti‐mouse or Alexa‐700 donkey anti‐rabbit secondary antibodies (ThermoFisher Scientific) and DAPI (Sigma‐Aldrich). Images and z‐stacks were acquired with a plan apochromat 40× oil objective on an SP8 inverted confocal microscope (Leica). Maximal intensity projections were generated with ImageJ (NIH) and normalized to DAPI pixel intensity.

### Mitotracker staining

PAECs and PASMCs were incubated with serum‐free ECM or DMEM media respectively containing 125 nM MitoTracker™ Orange CMTMros (ThermoFisher) for 25 min. Cells were washed three times in PBS (Sigma‐Aldrich) and then processed as fixed samples as described. Mitochondrial area and volume per cell were calculated using Volocity (Perkin‐Elmer) through Z‐stack analysis and thresholding. Nuclear volume was determined in Volocity by DAPI staining and used as a normalizing factor.

### Mito‐FerroGreen live‐staining

PAECs grown on imaging slides (Ibidi) were washed three times with HBSS supplemented with Ca^2+^ and Mg^2+^ (Sigma‐Aldrich) and incubated for 30 min with 5 µM Mito‐FerroGreen and Hoescht 3342 (Sigma‐Aldrich). Cells were then washed three times with HBSS supplemented with Ca^2+^ and Mg^2+^ and live‐imaged with an SP8 inverted confocal microscope microscope with a plan apochromat 40× oil objective (Leica). Mito‐FerroGreen pixels were determined with ImageJ (NIH).

### Mitosox live‐staining

PAECs were incubated with 5 µM Mitosox (Sigma‐Aldrich) and 5 µg/mL Hoescht 33342 (Sigma‐Aldrich) in HBSS with Ca^2+^ and Mg^2+^ (Life Technology) for 8 min, washed three times with HBSS with Ca^2+^ and Mg^2+^ before imaging with a LSM780 confocal microscope with a plan apochromat 63 × 1.4 NA oil objective (Zeiss). In some cases, cells were treated with 100 µM Deferoxamine (Sigma‐Aldrich) for 24 h before imaging. Mitosox and Hoescht 33342 integrated density was determined with ImageJ (NIH).

### Immunoflourescence analysis

Mitotracker CMTMros (chloromethyltetramethylrosamine) was measured to determine mitochondrial membrane potential (MMP). Its entry into mitochondrial is dependent upon the membrane potential and reduced fluorescence can indicate a reduction of this potential.^17^ Mitochondria retain these dyes regardless of fluctuation of MMP and so variation over time cannot be measured.^18^, ^19^ We have used this quantification here as an immediate measurement only. Cells were starved overnight and treated with hepcidin for 24 h. Then incubated with Mitotracker for 20 min at 37°C. Cells were then fixed and stained. Confocal z‐stacks were compiled as z projections according to pixel max intensity and measurements were taken in Volocity (Perkin‐Elmer). Fold change was calculated for average integrated density per cell relative to the control of that experiment.

### Cell cycle analysis

Cell cycle was investigated using a propidium iodide assay via flow cytometry following manufacturer's instructions (Invitrogen). Briefly, cells were washed in PBS, trypsinized, pelleted and resuspended in PBS with staining solution, incubated for 30 min in the dark and analyzed by flow cytometry (BD biosciences).

### Gene expression qPCR

Total RNA was extracted from cells using the RNAeasy Mini preparation Kit (Qiagen). Total RNA was measured by a Nano‐drop spectrophotometer. 0.1–0.5 μg of total RNA was used to produced cDNA, using MLV reverse transcriptase (Invitrogen), 10 mM dNTPs, oligo‐dT primers (Invitrogen) and RNase inhibitor (Applied Biosciences). Real‐time PCR using SYBR green (Sensi‐FAST lo‐ROX, Meridian Bioscience) was carried‐out on a Rotor‐Gene 6000 PCR machine. The change in expression was normalized to control, untreated samples.

### Statistics

Statistical analysis was performed on GraphPad (Prism). Graphs generated using GraphPad present data ± standard error of the mean (SEM) of the specified number of independent experiments. Some variability in results was observed and this appeared to be a donor effect.

## RESULTS

### Ferroportin and hepcidin are expressed and regulated in PAECs

**Figure 1:**
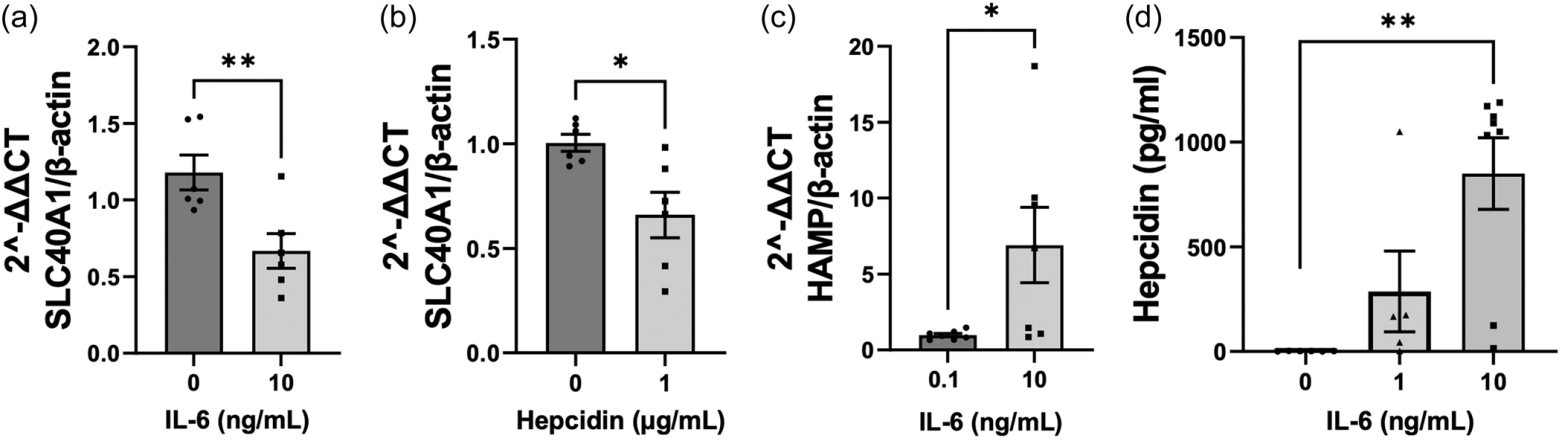
Ferroportin and hepcidin expression and regulation in pulmonary artery endothelial cell (PAEC) (a and b) quantification of ferroportin (SLC40A1) mRNA by RT‐qPCR in PAECs treated with 10 ng/mL IL‐6 (c) and 1 µg/mL hepcidin for 2 h, expressed as fold change of control (mean ± standard error of the mean [SEM]; *n* = 5 for (b) and *n* = 6 for (c), (c) hepcidin mRNA transcription in PAECs after IL‐6 stimulation for 2 h. Data shown are mean ± SEM *n* = 6. Unpaired *t*‐test was performed. *p < 0.05. (d) Quantification of ELISA specific to Hepcidin for media supernatants of PAEC untreated (control) or with 1–10 ng/mL IL‐6 for 24 h (mean ± SEM; *n* = 5–12); Student's *t* test performed for (b–d) and one‐way ANOVA with Bonferroni post hoc analysis for (d); *p < 0.05; **p < 0.01; ***p < 0.001.

In a recent study investigating ACE2 expression in PAECs we demonstrated the presence and modulation of the hepcidin‐ferroportin axis in this cell type by western blot and immunofluorescence.^20^ To corroborate and extend these initial findings we set out to determine cellular mRNA and protein production in PAECs in response to stimulation with hepcidin or IL‐6, known to regulate hepcidin expression.^21^, ^22^ L‐6 treatment of PAECs reduced ferroportin mRNA (Figure 1a), similar to the response seen following hepcidin treatment (Figure 1b). IL‐6 treatment of PAECs also induced hepcidin mRNA (HAMP) at 1 h (Figure 1c); variability was high in this response and appeared to be donor specific. Additionally, an increased cellular release of hepcidin protein was detectable in the media from PAECs treated with IL‐6 for 24 h (Figure 1d).

### Hepcidin treatment of hPAEC induces production of IL‐6, and augmented (autocrine) hepcidin release

**Figure 2:**
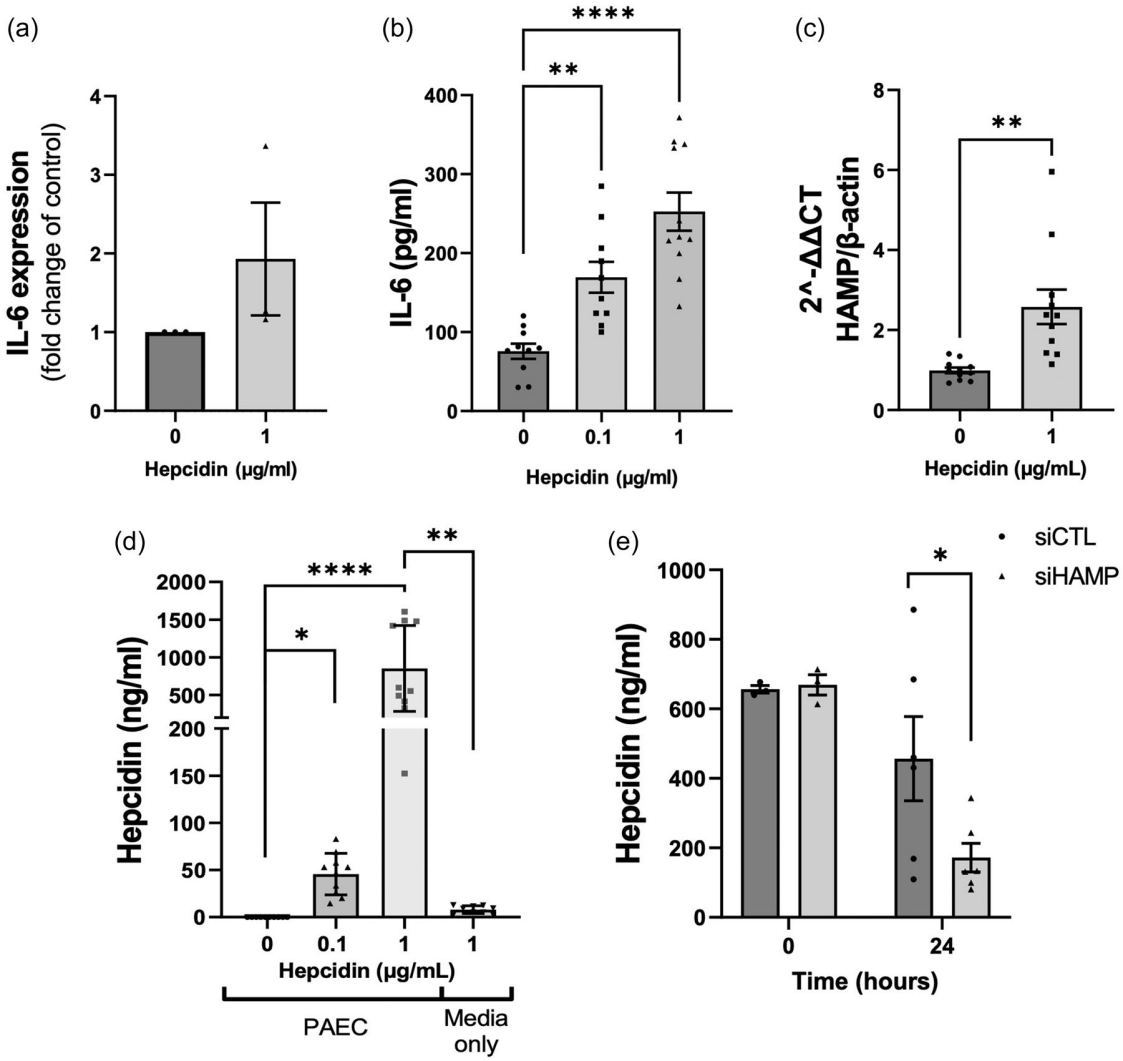
Hepcidin induces production of IL‐6 and hepcidin in pulmonary artery endothelial cell (PAEC). PAECs release IL‐6 after hepcidin treatment (a). IL‐6 mRNA transcription in PAECs after hepcidin stimulation for 5 h. Data shown are mean ± standard error of the mean (SEM) *n* = 3. (b) IL‐6 release into PAECs supernatants after 24 h stimulation with hepcidin (0.1, 1 µg/mL). PAECs supernatants were analysed by ELISA as described in section 2.6. Data shown are mean ± SEM *n* = 10. (c) Hepcidin mRNA transcription from PAECs after treatment with hepcidin for 1 h. Data shown are mean ± SEM *n* = 6. (d) ELISA analysis of hepcidin in media supernatants of PAEC untreated or with 0.1 or 1 µg/mL Hepcidin for 24 h. Control denotes media alone with 1 µg/mL Hepcidin for 24 h. Data shown are mean ± SEM *n* = 8 (e) ELISA analysis of hepcidin in supernatants of PAECs transfected with si‐Control (siCTL) or si‐HAMP (siHAMP) treated with 1 µg/mL Hepcidin at time 0 (6 h after transfection) and 24 h after transfection. Data shown are mean ± SEM; siCTL, *n* = 3; siHAMP, *n* = 6. Unpaired *t*‐test (a, c, and e) one‐way ANOVA with Bonferroni post hoc analysis was performed (b and d); ****p < 0.0001; **p < 0.01; *p < 0.05.

Hepcidin treatment of PAECs resulted in elevated levels of IL‐6 mRNA in comparison to untreated controls within 1 h of exposure (Figure 2a). In addition, a significant increase of IL‐6 protein release from PAECs was detected in media from hepcidin treated PAECs by 24 h, as determined by ELISA (Figure 2b).

Interestingly, treatment of PAECs with hepcidin appeared to induce augmented (autocrine) release of hepcidin. Evidence to support this includes the following: First, a significant increase in HAMP mRNA was observed at 1 h (Figure 2c), followed by significant release of hepcidin into the media compared to control over 24 h, as determined by ELISA (Figure 2d). Second, media alone, but with the same amount of hepcidin added (Figure 2d), resulted in low levels of hepcidin relative to that seen with cells present. Third, 0.1 and 1 µg/mL hepcidin treatment both induced significant hepcidin release by cells into the media compared to cells where no hepcidin was added or to media alone with hepcidin addition (Figure 2d). Furthermore, this response was shown to increase over time, up to 24 h (Figure S2A). Fourthly, comparison between cells incubated with hepcidin and cell only controls reinforced the role of hepcidin as mediator for subsequent hepcidin release from PAECs (Figure 2d). Finally, downregulation of hepcidin (HAMP) through transfection of PAEC with targeting siRNA revealed a significant reduction in hepcidin present in the media of hepcidin treated cells compared with a control siRNA (Figure 2e).

Hepcidin treatment in PAECs also caused significantly increased release of monocyte chemoattractant protein‐1 (MCP‐1, Figure S2B), a key chemokine regulating migration and infiltration^23^ as well as IL‐8 (Figure S2C).

### Hepcidin induces mitochondrial dysfunction in PASMCs but not in PAECs

To investigate if responses to hepcidin treatment and associated cellular iron retention within PAECs influenced mitochondrial function, cells were given direct hepcidin treatments for 24 h. PASMCs were similarly challenged to determine variations between both cell types.

#### Morphological changes

**Figure 3:**
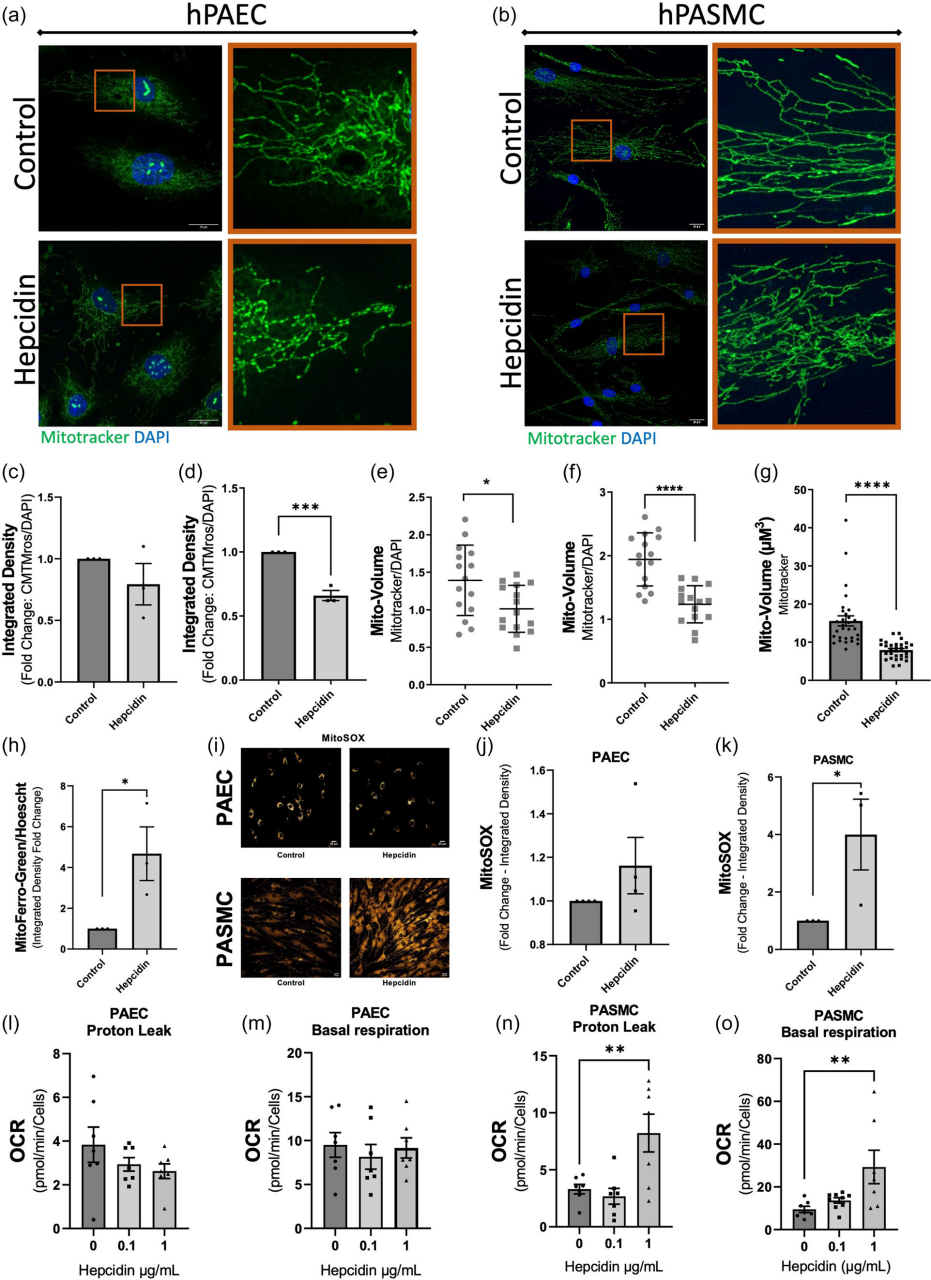
Hepcidin treatment produces mitochondrial effects in pulmonary artery endothelial cell (PAEC) and pulmonary artery smooth muscle cells (PASMC). PAEC (a) and PASMC (b) were stained with Mitotracker (green) and DAPI (blue) following treatment with or without hepcidin. Orange boxes show magnified region. Scale bars, 20 µm. (c) Integrated density of PAEC Mitotracker was quantified and represented as average fold change relative to control for multiple cells per condition (*n* = 3). (d) Integrated density of PASMC Mitotracker was quantified and represented as fold change relative to control as with c (*n* = 3). (e) Mitochondrial volume of PAEC cells following treatment with or without hepcidin. Mitochondrial volume normalized to nuclear (DAPI) volume (*n* = 3 independent experiments, 15 cells total). (f) Mitochondrial volume of PASMC following treatment with or without hepcidin. Mitochondrial volume normalized to nuclear (DAPI) volume (*n* = 3 independent experiments, 15 cells total). (g) Mitochondrial volume (μM^3^), determined using mitotracker for PASMC following treatment with or without hepcidin (*n* = 3 independent experiments, 30 cells total). (h) Fold change of integrated density for MitoFerro green normalized to Hoescht for PASMC (mean ± standard error of the mean [SEM]; *n* = 3). (I) Representative images of PAEC and PASMC treated with or without hepcidin and stained with MitoSOX. (j) Quantification of MitoSOX integrated density for PAEC shown as fold change hepcidin versus control (mean ± SEM; *n* = 4). (k) Quantification of MitoSOX integrated density for PAEC shown as fold change hepcidin versus control (mean ± SEM; *n* = 3). (l) Proton leak and (m) basal respiration from PAEC treated with hepcidin. Oxygen consumption rate (OCR) was determined by Seahorse XF analyser mito stress test (*n* = 7). (n) Proton leak and (o) basal respiration from PASMC treated with hepcidin (*n* = 7). Students *t*‐test was performed or one‐way ANOVA with Bonferroni post hoc analysis for (l–o). Mean ± SEM for all bar charts. ***p < 0.001; **p < 0.01, *p < 0.05.

In PAECs, mitochondrial networks appeared to bud and became disrupted following hepcidin treatment, but no fractionation was evident (Figure 3a). By contrast, mitochondria in PASMCs exposed to hepcidin demonstrated obvious changes in morphology as strikingly defined networks became fractionated (Figure 3b). In addition, PASMC mitochondria showed no budding in contrast to PAECs but did appear less uniform in their arrangement following hepcidin treatment (Figure 3b). Because Mitotracker Orange (CMTMros) enters mitochondria based on MMP,^24^ fluorescence can infer an immediate measurement of MMP; however, this is not a reliable metric over time.^18^ Whilst no change to fluorescence was revealed in hepcidin treated PAECs (Figure 3c), it was significantly reduced in similarly treated PASMCs (Figure 3d) indicative of a reduction in MMP. This observation was supported by subsequent seahorse experiments.

Moreover, PAECs showed reduced ( ~25%) mitochondrial volume per cell in response to hepcidin treatment (Figure 3e) whereas in PASMCs, a much more obvious reduction in per cell mitochondrial volume following 24 h hepcidin treatment of ~50% was evident (Figure 3f). For these analyses mitochondrial volume was normalized per cell against nuclear volume to account for variations in cell size. Importantly, in this regard, nuclear volume showed no change in response to hepcidin treatment at 24 h in PASMCs (Figure S3A). Furthermore, the change in mitochondrial volume observed were confirmed by analysing mitochondrial volume using the mitochondrial marker TOM20, which revealed significant reductions on average mitochondrial volume in PASMCs following 24 h hepcidin treatment (Figure S3B).

#### Fragmentation of mitochondrial networks were observed in PASMCs

This was quantified as a change on average mitochondrial volume which revealed smaller detectable units. A larger average indicates the continuation of a network and smaller average is indicative of a broken, fragmented network. Treatment of PASMCs with hepcidin for 24 h caused a significant reduction on average mitochondrial volume (Figure 3g).

#### Mitochondrial iron retention and ROS production

Changes to mitochondrial iron levels were determined by use of Mito‐FerroGreen, a mitochondria‐specific iron probe that becomes fluorescent when reacting with labile mitochondrial Fe^2+^.^25^ In PAECs incubated with hepcidin, increase in mitochondrial specific iron as determined by increases in mito‐ferrogreen staining (Figure S3C), was expressed as integrated density (Figure 3h). It proved technically challenging to use this technique in PASMCs. However, we have previously reported PASMC iron accumulation in response to hepcidin challenge although mitochondrial specific iron determinations were not undertaken in that study.^11^

To further investigate cell specific mitochondrial responses, assessment of mitochondrial ROS production was undertaken using the mitochondrial specific dye, MitoSOX, which becomes fluorescent upon superoxide‐mediated oxidation. There was no noticeable change in MitoSOX staining following hepcidin treatment in PAECs but an obvious response was observed in PASMCs (Figure 3i–k).

Mitochondrial function was further investigated through seahorse assay. Proton leak measures the number of protons entering the extracellular space, indicating changes in membrane potential and membrane function. Increases in proton leak indicate mitochondrial damage.^26^ Basal respiration describes the oxygen consumption used to meet cellular ATP demand resulting from mitochondrial proton leak. Changes indicate energetic demand of cells under baseline conditions. Hepcidin treatment revealed little change in dynamics of PAECs as measured by seahorse, with a slight decrease in proton leak (Figure 3l) and a very slight decrease in basal respiration (Figure 3m). By contrast, there was a significant increase in proton leak (Figure 3n) and basal respiration (Figure 3o) for PASMCs treated with 1 µg/mL hepcidin.

### Conditioned media from hepcidin treated PAECs induces migration, proliferation and mitochondrial responses in PASMCs

#### Ferroportin expression

**Figure 4:**
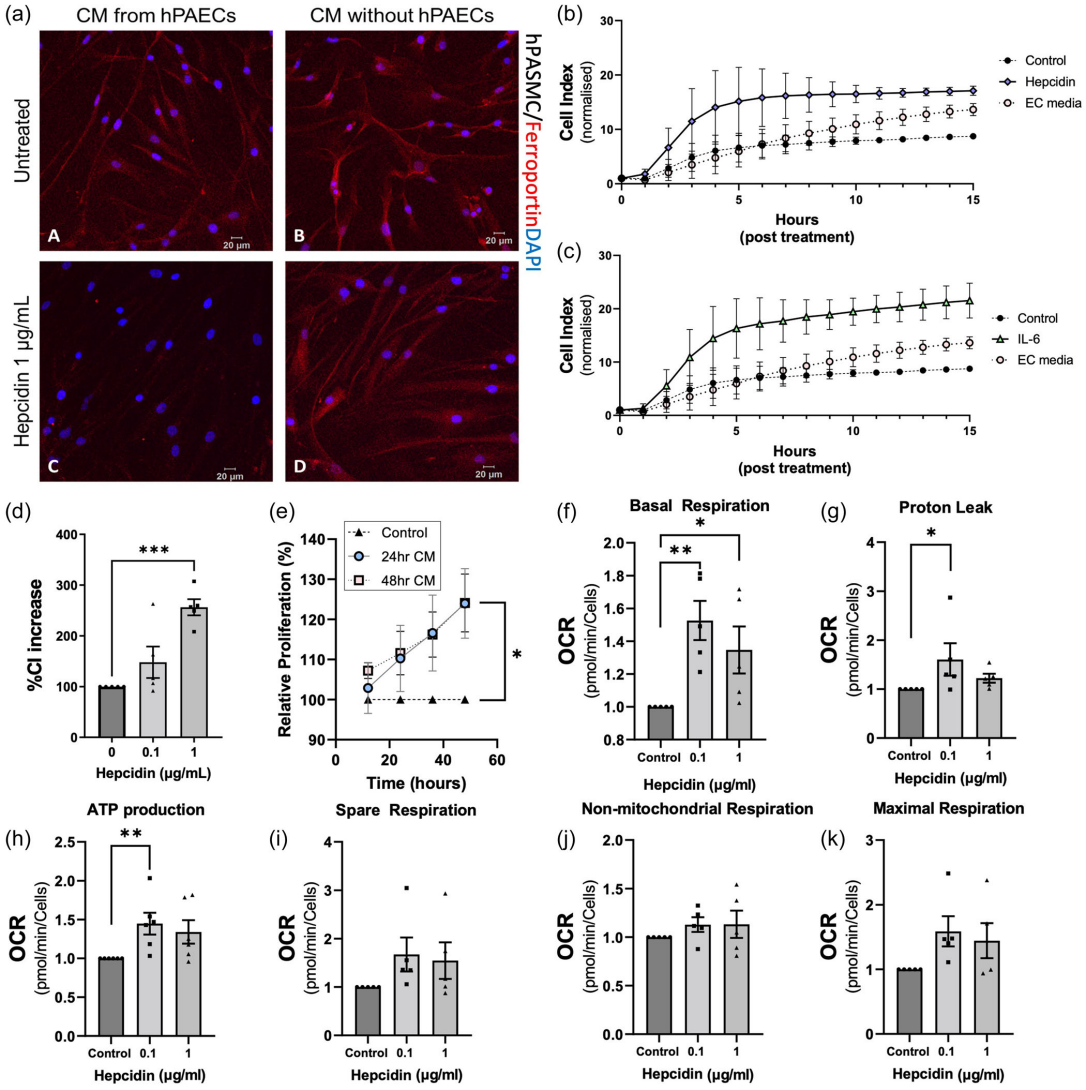
Effects of pulmonary artery endothelial cell (PAEC) conditioned media treated with hepcidin on hPASMC. Immunoflourescent images of pulmonary artery smooth muscle cells (PASMC) expression ferroportin after 24 h exposure to PAEC conditioned media (CM). CM from PAEC indicates presence of PAEC with or hepcidin and CM without PAEC is media alone with or without hepcidin (a). Ferroportin (red) and DAPI (blue). Changes in pulmonary artery smooth muscle cell migration in hepcidin‐treated conditioned media (b) and IL‐6 treated conditioned media (c) or endothelial cell (EC) media alone measured by xCELLigence assay. (d) Quanitifation of PASMC migration at 8 h with treatment of conditioned media treated with hepcidin. (e) Initial PASMC proliferation in response to 24 and 48 h conditioned media with or without hepcidin addition, measured by xCELLigence assay. (f–k) PASMC mitochondria metabolism changes after 24 h treatment with PAECs conditioned media. Oxygen consumption rate (OCR) was determined by Seahorse XF analyser mito stress test. All data shown are mean ± standard error of the mean (SEM) *n* = 6. Kruskal‐Wallis test followed by Dunn's post hoc test was performed for all but e, where two‐way ANOVA with Tukey post hoc analysis was performed *p < 0.05; **p < 0.01, *** *p* < 0.001.

PASMCs incubated for 24 h with conditioned media, obtained from PAECs following 24 h of treatment with hepcidin (1 µg/mL), resulted in a complete loss of ferroportin staining as determined by confocal imaging (Figure 4a). By contrast, media from untreated PAECs or “hepcidin in media only” controls did not alter ferroportin staining (Figure 4a). These results suggested that an endothelial derived component, possibly hepcidin, (see Figure 2d) was responsible. Given these findings, relevant functional responses in relation to the conditioned media were then assessed in PASMCs.

#### PASMC migration using the xCELLigence real time cell analyser (RTCA) system

24 h conditioned media either from IL‐6 or hepcidin challenged PAECs caused significant PASMC migration (Figure 4b,c). This was significant by 8 h of exposure compared to untreated PAEC conditioned media controls for 1 ng/mL of IL‐6 (Figure S4A) and 1 µg/mL hepcidin (Figure 4b,d and S4A). Conditioned media from 0.1 to 1 µg/mL hepcidin treatments revealed concentration specific increases in this migratory response in PASMCs at 8 h (Figure 4d). Additional studies using direct hepcidin treatments at the same concentrations in media alone did not cause migration, so excluding the possibility that these agents were acting directly as proliferative agents (Figure S4B) and confirming that the migratory responses observed were in the PAEC conditioned media. Furthermore, PAEC control media (no hepcidin pretreatment) did not induce this migratory response (Figure S4A). Moreover, PASMC migration increased to a significant and even greater extent when cells were exposed to 24 h conditioned media. However, when using 48 h conditioned media, the migratory response had diminished. (Figure S4C).

#### PASMC proliferation using the xCELLigence RTCA system

Cell index measurements revealed a significantly increased initial rate of proliferation in response to treatment with 48 h PAEC conditioned media but not with 24 h conditioned media (Figure S4D). In addition, as cell index measurements are made by the xCelligence RTCA system continuously, in real time, over a period of 48 h, assessment of total proliferation was also undertaken. Analysis of data was limited to 4 time points (12, 24, 36, and 48 h). Overall, conditioned media significantly increased the total proliferation of PASMCs after 48 h (Figure 4e). Conditioned media from 24 h treated PAECs increased total 48 h proliferation of PASMCs by 24% (95% CI = 1.89–46.1, *p*< 0.05), and conditioned media from 48 h treated PAECs by 24% (95% CI = 0.256–47.9, *p* < 0.05). No significant effect of conditioned media was seen at 12, 24, or 36 h time points but there was an observable trend of proliferation increasing with time (Figure 4e).

#### Cell cycle by flow cytometry

To further investigate cellular state in PASMCs in response to direct treatment with hepcidin or IL‐6, cell cycle was investigated with flow cytometry. Hepcidin at 1 µg/mL induced a significant reduction in cells in G0/G1 phase and an increase in the growth phases, S and G2/M (Figure 4e). A similar effect was shown with 10 ng/mL IL‐6 at 24 h with increase in cells in S and G2/M (Figure S4F).

Proliferation of PAECs was also assessed at 24 h for direct treatment of hepcidin and IL‐6 with BrdU assay. At 24 h, hepcidin challenge revealed no change in proliferation versus control (Figure S4G) whereas IL‐6 treatment revealed a significant increase in proliferation relative to control (Figure S4H). Cell cycle analysis of PAECs following 24 h treatment with hepcidin or IL‐6 revealed no changes compared to control cells (Figure S4I,J).

PAEC viability was investigated using MTS assay. Direct treatment of hepcidin revealed a slight but insignificant increase in viability (Figure S4K) whereas 10 ng/mL of IL‐6 treatment increased viability significantly compared to control (Figure S4L). Further to this, cell death at varying concentrations up to 1 µg/mL hepcidin and 10 ng/mL IL‐6 was investigated with Alarmar blue assay and no significant changes were observed (Figure S4M,N).

#### Mitochondrial respiration

Given the effects of conditioned media on ferroportin expression seen in PASMCs (Figure 4a) and the studies presented in Figure 3, which demonstrated considerable levels of mitochondrial dysfunction in PASMCs after direct treatments with hepcidin, further investigations of mitochondrial respiration were undertaken to assess responses to conditioned media. PASMCs treated with 24 h conditioned media demonstrated significant changes in basal respiration (Figure 4f), proton leak (Figure 4g) and ATP production (Figure 4h). Increases in spare respiration (Figure 4i), nonmitochondrial respiration (Figure 4j) and maximal respiration (Figure 4k) were also observed. Further to this, the effects of 48 h conditioned media upon PASMCs mitochondrial respiration was also investigated. In contrast to the effects of 24 h conditioned media, maximal respiration and spare respiration were both significantly reduced (Figure S5A,B). Basal respiration, proton leak and nonmitochondrial respiration also showed relative reduction compared to control (Figure S5C–E). Together these data present a considerable change to PASMC metabolism in response to hepcidin treated PAEC conditioned media treatment.

Given that hepcidin has previously been shown to increase proliferation in PASMCs^11^ and that studies presented in Figure 2 and described above indicate that hepcidin treatment of PAECs caused greatly augmented cellular release of hepcidin, it seems plausible to hypothesize a causative link between PAEC hepcidin release and PASMCs proliferation, outlined in the graphical abstract.

## DISCUSSION

This research extends our understanding of the hepcidin‐ferroportin axis in human PAECs and PASMCs, providing novel observations of the modulation of this axis both at intracellular and intercellular levels. Novel findings include: 1, treatment of human PAECs with hepcidin induced production of hepcidin (in an autocrine fashion), IL‐6 and other cytokines including MCP‐1 and IL8. 2: Direct treatment of PAECs and PASMCs with hepcidin alone generated contrasting mitochondrial stress, whereby PAECs exhibited less mitochondrial stress than PASMCs. 3: Treatment of PASMCs with conditioned media from PAECs treated with hepcidin induced proliferative, migratory, and metabolic mitochondrial changes. The studies presented here confirm a key regulatory function for expression and modulation of the hepcidin‐ferroportin axis within cells of the pulmonary vasculature. Moreover, crosstalk between PAECs and PASMCs indicates a potential paracrine role for hepcidin (and other associated molecules) in this setting.

### Modulation of the hepcidin‐ferroportin axis in PAEC and PASMC

There are few studies demonstrating the presence of ferroportin in endothelial cells although it has been described in brain, cardiac and retinal cells.^27^, ^28^, ^29^ In this paper, we have clearly demonstrated the presence of ferroportin in PAECs and its regulation in response to hepcidin and IL‐6 treatment. Furthermore, we observed upregulation of hepcidin mRNA and hepcidin in the media of PAECs treated with IL‐6. These findings confirm and expand our preliminary observations in PAECs^20^ by demonstrating modulation of this axis in these cells in a similar fashion to that previously published by our group in PASMCs.^11^

IL‐6 has been implicated as a potential mediator in PAH^30^, ^31^ but a recent clinical trial using the IL‐6 receptor antagonist, tocilizumab, failed to report any benefit in PAH patients.^32^ In contrast, we present a potential role for IL‐6 modulation of the hepcidin‐ferroportin axis and proliferation in PAECs. However, IL‐6 is not the only positive regulator of this axis; interferon alpha^33^ and IL‐1^21^ amongst others, also up‐regulate hepcidin production in cells types more traditionally associated with iron homeostasis; as yet no evaluations have been undertaken in PAECs. Our ongoing research further demonstrates the ability of PAECs to promote IL‐6 gene transcription and release in response to hepcidin exposure, expanding the notion of a localized regulation of iron homeostasis in these cells.

Unexpectedly, it was also observed that hepcidin treatment significantly enhanced hepcidin (HAMP) mRNA production and hepcidin release from PAECs over time. The observation of potential de novo synthesis by PAECs was further supported by siRNA knockdown studies of HAMP. Additionally, comparative studies with cell free media containing hepcidin, incubated using the standard cell culture protocol, showed a dramatic loss of hepcidin signal over time supporting the concept of endogenous (autocrine) production by PAECs rather than any artefact of treatment dose; it is established that hepcidin has a short half‐life in biological matrices.^34^ However, to finally determine that (dosage) hepcidin was a contributing factor, buffered saline containing hepcidin was incubated under the same conditions as cells and over the same time frame, there was a rapid loss in measured hepcidin over the time course to undetectable levels (hence data is not shown). This result strongly indicates that media related binding effects, which could be reversed by conditional changes over time, so releasing hepcidin and providing the enhanced signal seen, were invalid. Having therefore excluded artefactual effects, the study clearly indicates the involvement of PAECs in hepcidin production and release, demonstrating the potential for localized autocrine regulation of the hepcidin‐ferroportin axis in these cells.

### Hepcidin modulates mitochondrial and respiratory function in PAEC and PASMC

As modulation of the hepcidin‐ferroportin axis likely impacts cellular iron resources and fluxes potentially impacting mitochondrial function, we investigated mitochondrial function in both PASMCs and PAECs following hepcidin treatment. Confocal imaging and analysis of mitochondrial networks showed distinct changes in mitochondrial morphology and membrane potential for both cell types following hepcidin treatment; however, it was apparent that PAEC mitochondria were more resistant to hepcidin treatment than PASMC mitochondria. Additionally, mitochondrial reactive species production was apparent in PASMCs but not replicated in PAECs.

A role for mitochondrial iron‐loading is well established in health and disease.^35^, ^36^, ^37^ Importantly, mitochondrial responses described here are similar to responses observed in mesenchymal stromal cells where iron overload resulted in mitochondrial fragmentation in a ROS dependant manner.^38^

Increased mitochondrial iron retention was observed in PAECs in response to hepcidin. Technical issues with mitoferro‐green prevented comparative examination in PASMCs but we have previously reported iron loading in PASMCs in response to hepcidin.^11^ Hepcidin treatment produced observable mitochondrial respiratory dysfunction assessed by seahorse assay, presumably through iron retention, in a more significant manner in PASMCs than PAECs. Changes in respiration may suggest a shift to glycolytic metabolism, previously reported to be linked to aspects of disease pathology in PAH.^39^ Together, our reported mitochondrial studies clearly demonstrated that mitochondria of PAECs are impacted less by modulation by the hepcidin‐ferroportin axis than PASMCs.

### Media from PAECs treated with hepcidin induces proliferative, migratory and respiratory changes in PASMCs

Our data indicates that PAECs produce and release significant amounts of hepcidin in response to treatment with hepcidin. To explore the potential relationship of the hepcidin‐ferroportin axis between PAECs and PASMCs, conditioned media from PAECs treated with hepcidin was exposed to PASMCs. A striking, almost complete, loss of ferroportin staining was apparent in PASMCs treated this way when compared to untreated PAEC conditioned media. Furthermore, media containing hepcidin and conditioned without PAECs over the same incubation time frame was unable to cause loss of ferroportin staining as was hepcidin free media. These results indicate the necessity for PAEC involvement and are indicative of endogenous hepcidin production by PAECs. In addition, the conditioned media was able to induce significant migratory response in PASMCs and a significant proliferative response when media was conditioned for 48 h.

Pronounced changes in mitochondrial respiration were observed with 24 and 48 h conditioned media. We were unable to find an intervention to specifically target hepcidin in conditioned media, but PAEC conditioned media controls lacking hepcidin were unable to replicate findings seen with PAEC hepcidin conditioned media, which strongly suggests that hepcidin produced by PAECs is the active component facilitating mitochondrial dysfunction and PASMC proliferation.

Indeed, mitochondria are known to be involved in aspects of cell fate including proliferation.^40^, ^41^ As for the rapid and extensive PASMC migratory responses observed in PASMC exposed to PAEC hepcidin conditioned media, it is not obvious how hepcidin would facilitate this response as there is no literature to our knowledge indicating that hepcidin has chemotactic properties. Moreover, hepcidin alone in media was unable to cause migration of PASMCS reinforcing this notion. However, significantly elevated levels of cytokines were also seen in such conditioned media and may well offer a plausible explanation for the migratory responses observed. MCP‐1 and IL‐8 are both known to possess chemoattractant properties^23^, ^42^ including with relevance for pulmonary vascular migratory responses in PAH.^43^, ^44^, ^45^ Based on our findings the proposed mechanism for PAH progression at the level of the pulmonary vasculature is best illustrated in the graphical abstract.

## SUMMARY

These studies have demonstrated the effective modulation of a hepcidin‐ferroportin iron regulatory axis in PAECs. It is also apparent that PAECs are more tolerant of exposure to hepcidin as evidenced by limited changes to mitochondrial morphology, ROS production and respiration in comparison to that seen with PASMCs. The pronounced effects of conditioned media from PAECs on PASMC function, including loss of ferroportin expression, altered mitochondrial respiration and ultimately enhanced proliferation, strongly implicate hepcidin released from PAECs as the driver of these responses. It is likely that enhanced iron retention in PASMCs due to loss of ferroportin mediated iron export, stimulated proliferation, both via mitochondrial related responses in addition to more classically recognized mechanisms.

Further evaluation of the hepcidin‐ferroportin axis under low oxygen tensions, with specific sensitive measures of cellular iron pools and in relevant in vivo models of PAH may well offer additional insight. This would strengthen the case for therapeutic intervention to target this axis in patients with PAH as would more in‐depth studies of any role for IL‐6 in these including the use of blocking antibodies.

## AUTHOR CONTRIBUTIONS

*Conceptualization:* Theo Issitt, Quezia K. Toe, Gregory J. Quinlan, and S. John Wort. *Data curation*: Theo Issitt, and Quezia K. Toe. *Formal analysis*: Theo Issitt and Quezia K. Toe. *Funding acquisition*: Gregory J. Quinlan and S. John Wort. *Investigation*: Theo Issitt, Quezia K. Toe, Sofia L. Pedersen, Thomas Shackshaft, Maziah Mohd Ghazaly, Laura West, Nadine D. Arnold, Abdul Mahomed, George W. Kagugube, and Latha Ramakrishnan. *Methodology*: Theo Issitt, Quezia K. Toe, Gregory J. Quinlan, and S. John Wort. *Project administration*: Allan Lawrie, Gregory J. Quinlan, and S. John Wort. *Resources*: Gregory J. Quinlan. *Visualization*: Theo Issitt. *Writing—original draft*: Theo Issitt. *Writing—review and editing*: Allan Lawrie, Gregory J. Quinlan, and S. John Wort. All authors have read and agreed to the published version of the article.

## CONFLICT OF INTEREST STATEMENT

The authors declare no conflict of interests.

## ETHICS STATEMENT

The use of normal lung tissue has been approved by the Royal Brompton and Harefield NHS Trust Research Ethics Committee (ethics number GQJW1). All procedures were carried out in accordance with the relevant guidelines and regulations. All patients gave written, informed consent before the use of their lung tissue.

## Supporting information

Supporting information.

Supporting information.

Supporting information.

Supporting information.

Supporting information.
