# Supplementary figures and images for: The hepcidin‐ferroportin axis influences mitochondrial function, proliferation, and migration in pulmonary artery endothelial and smooth muscle cells
Source: Pulm Circ. 2024 Dec 18;14(4):e70006. doi: 10.1002/pul2.70006 (PMC11653027; doi:10.1002/pul2.70006)

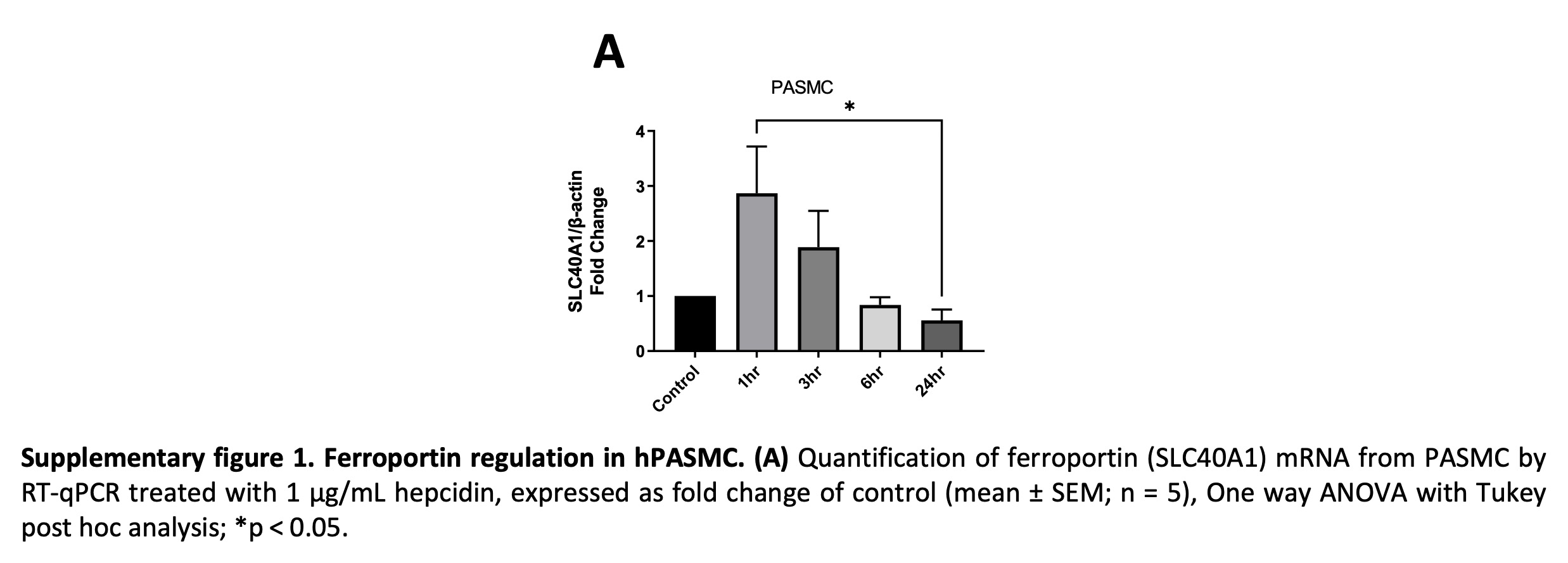

Supplement: Supplementary file 1 — Supporting information. [file PUL2-14-e70006-s005.jpg]

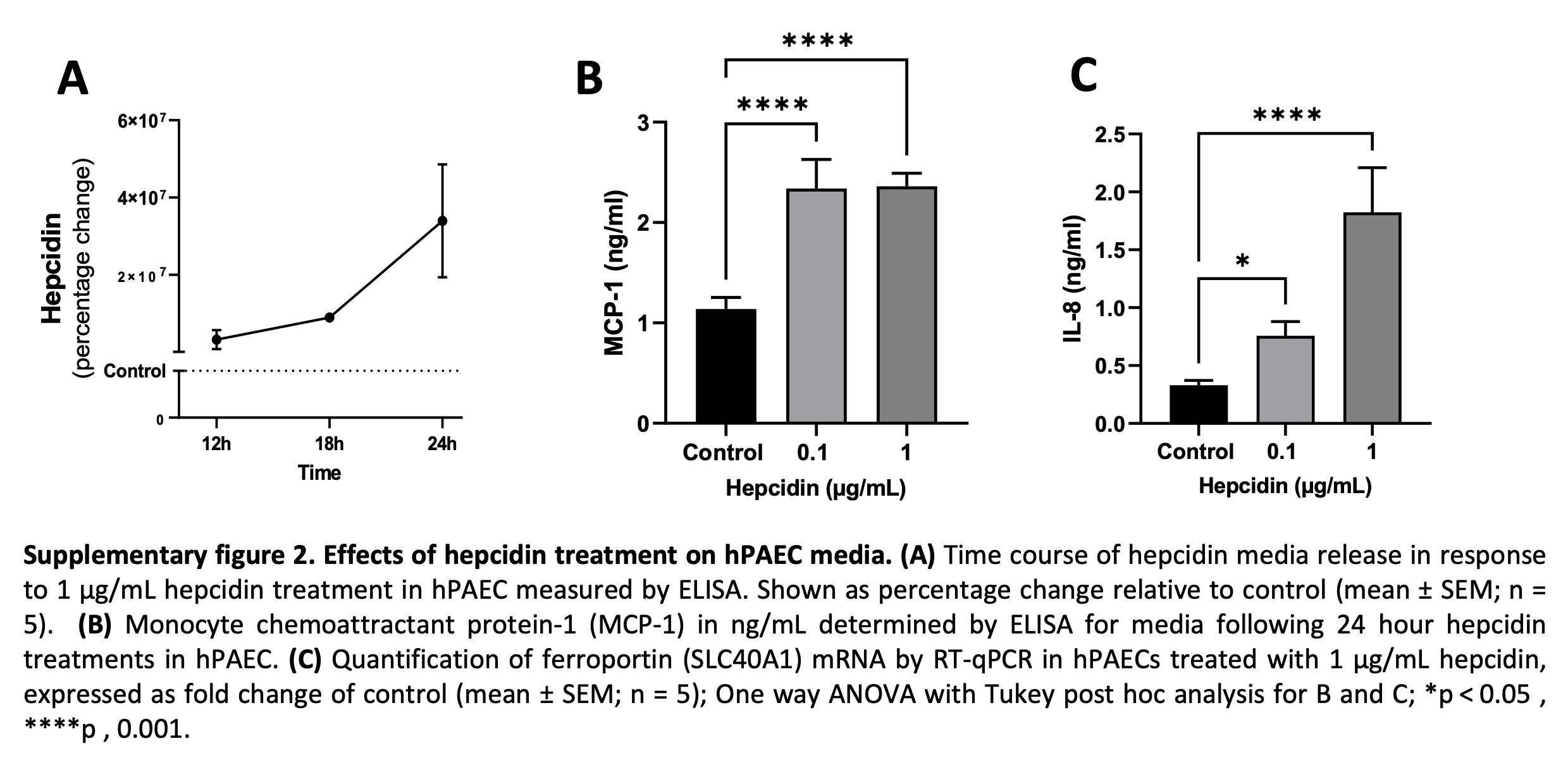

Supplement: Supplementary file 2 — Supporting information. [file PUL2-14-e70006-s004.jpg]

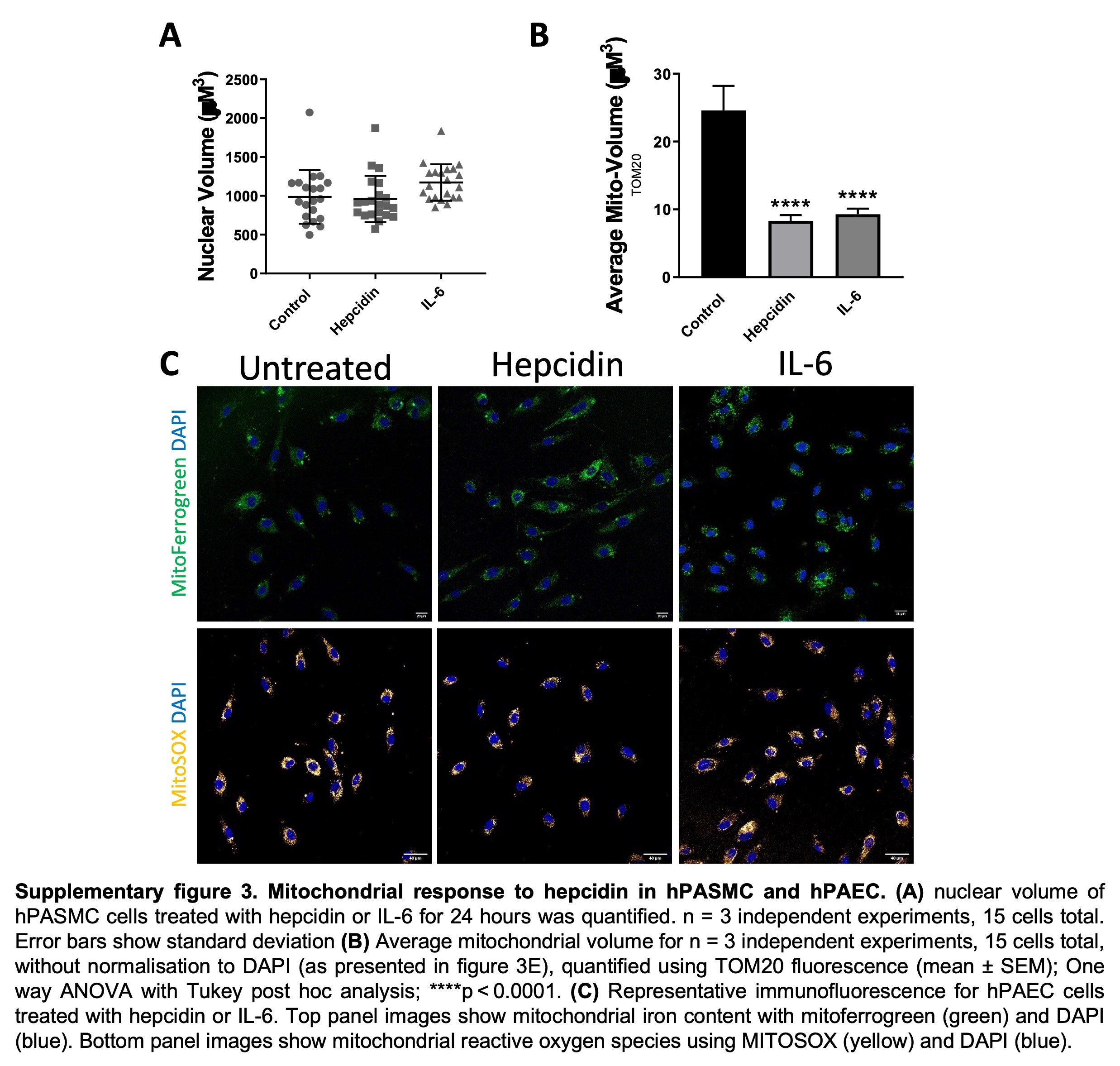

Supplement: Supplementary file 3 — Supporting information. [file PUL2-14-e70006-s002.jpg]

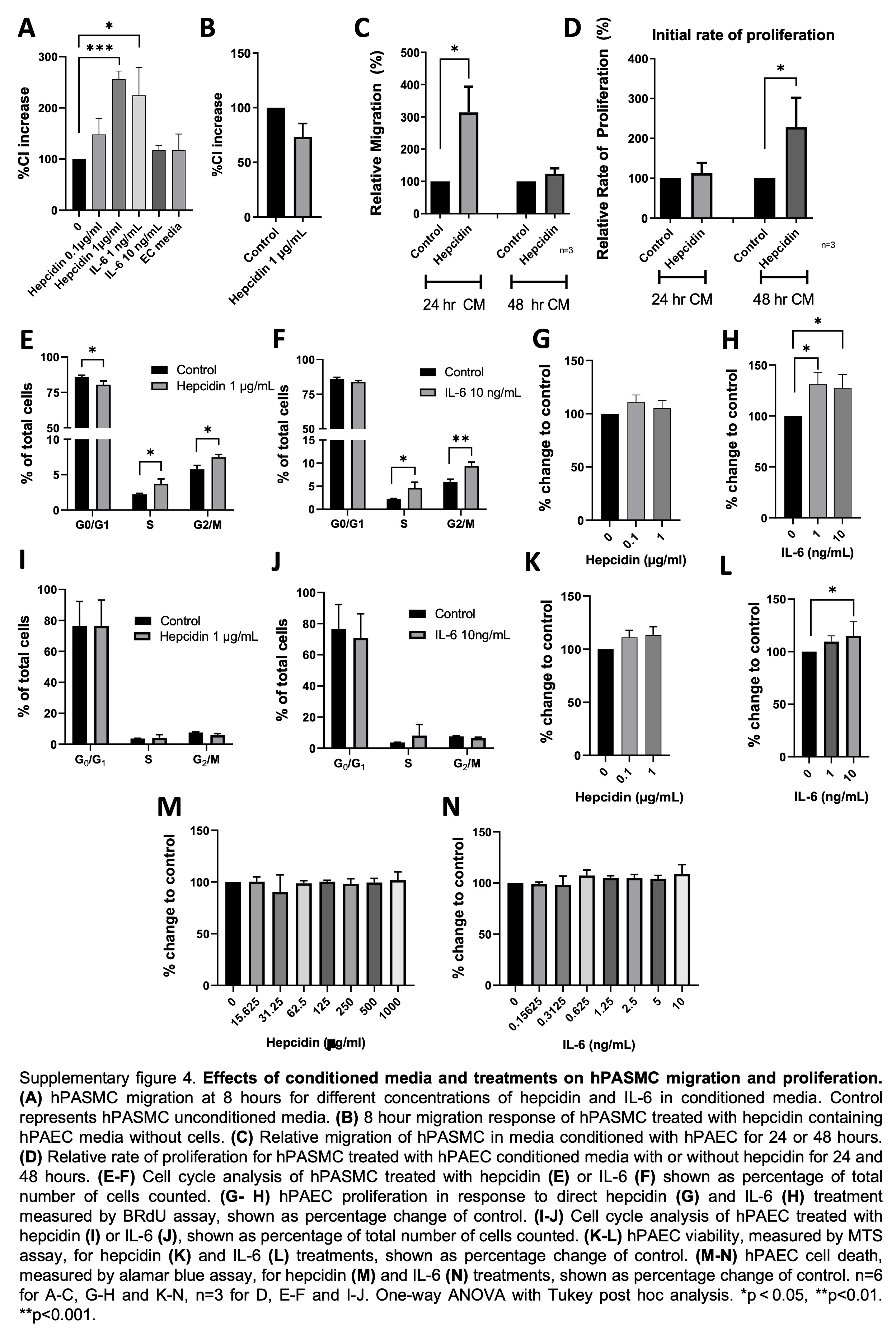

Supplement: Supplementary file 4 — Supporting information. [file PUL2-14-e70006-s001.jpg]

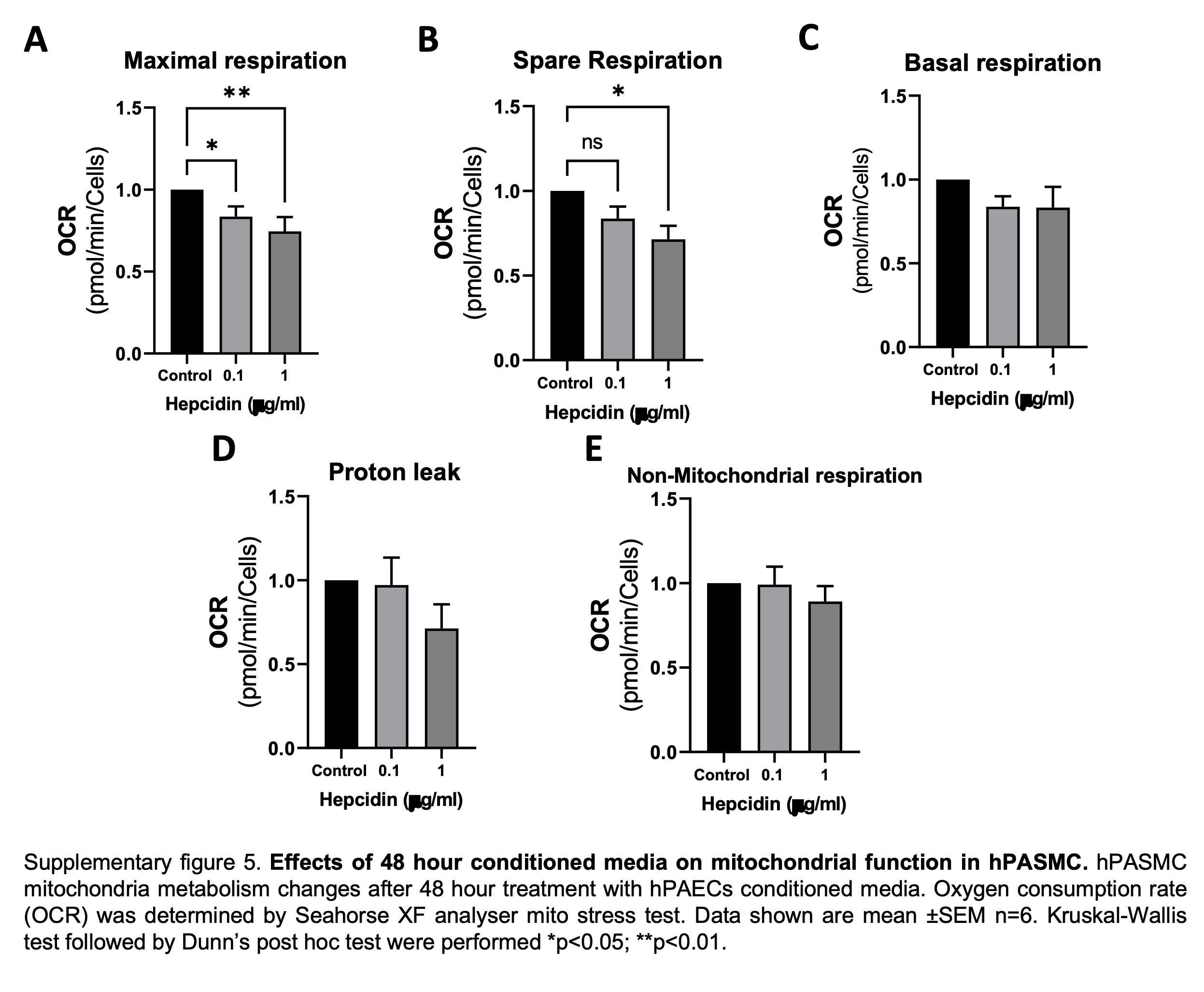

Supplement: Supplementary file 5 — Supporting information. [file PUL2-14-e70006-s003.jpg]
